# Structure and Metabolically Oriented Efficacy of Fucoidan from Brown Alga *Sargassum muticum* in the Model of Colony Formation of Melanoma and Breast Cancer Cells

**DOI:** 10.3390/md21090486

**Published:** 2023-09-10

**Authors:** Roza V. Usoltseva, Anastasiya O. Zueva, Olesya S. Malyarenko, Stanislav D. Anastyuk, Olga P. Moiseenko, Vladimir V. Isakov, Mikhail I. Kusaykin, Airong Jia, Svetlana P. Ermakova

**Affiliations:** 1 G.B. Elyakov Pacific Institute of Bioorganic Chemistry, Far Eastern Branch of the Russian Academy of Sciences, 159, prosp. 100 Let Vladivostoku, Vladivostok 690022, Russia; zstasya95@gmail.com (A.O.Z.); malyarenko.os@gmail.com (O.S.M.); sanastyuk@piboc.dvo.ru (S.D.A.); svetlana_ermakova@hotmail.com (S.P.E.); 2 Key Laboratory for Applied Microbiology of Shandong Province, Biology Institute of Shandong Academy of Sciences, Jinan 250014, China; jiaar@sdas.org

**Keywords:** *Sargassum muticum*, fucoidan, 2-deoxy-d-glucose, MDA-MB-231, SK-MEL-28 cells, glycolysis, anticancer activity

## Abstract

This work reports the detailed structure of fucoidan from *Sargassum miticum* (2SmF2) and its ability to potentiate the inhibitory effect of glycolysis inhibitor 2-deoxy-d-glucose (2-DG). 2SmF2 was shown to be sulfated and acetylated galactofucan containing a main chain of alternating residues of 1,3- and 1,4-linked α-l-fucopyranose, fucose fragments with monotonous 1,3- and 1,4-type linkages (DP up to 3), α-d-Gal-(1→3)-α-L-Fuc disaccharides, and 1,3,4- and 1,2,4-linked fucose branching points. The sulfate groups were found at positions 2 and 4 of fucose and galactose residues. 2SmF2 (up to 800 µg/mL) and 2-DG (up to 8 mM) were not cytotoxic against MDA-MB-231 and SK-MEL-28 as determined by MTS assay. In the soft agar-based model of cancer cell colony formation, fucoidan exhibited weak inhibitory activity at the concentration of 400 µg/mL. However, in combination with low non-cytotoxic concentrations of 2-DG (0.5 or 2 mM), 2SmF2 could effectively inhibit the colony formation of SK-MEL-28 and MDA-MB-231 cells and decreased the number of colonies by more than 50% compared to control at the concentration of 200 µg/mL. Our findings reveal the metabolically oriented effect of fucoidan in combination with a glycolysis inhibitor that may be beneficial for a therapy for aggressive cancers.

## 1. Introduction

Despite progress in cancer therapy and a huge number of clinical trials of new drugs, cancer is still the second leading cause of death after cardiovascular disease in developed countries [1].

It is well known that the metabolism of malignant cells differs significantly from that of normal cells. Thus, cancer cells are prone to accelerated glucose uptake for anaerobic glycolysis, which is their preferred way of energy production [2]. Therefore, the search for new inhibitors of cancer cell metabolism is a promising field in anticancer research [3].

It has been reported that interference with glycolysis by the non-metabolizable glucose analogue 2-deoxy-D-glucose (2-DG) can effectively inhibit glycolysis and lead to cancer cell death [4,5]. On the other hand, daily administrations of large doses of 2-DG over a long period were found to cause toxicity, diaphoresis, hypoglycemia, and disturbances of the central nervous system, which limited the potential of 2-DG application as a single therapeutic agent [6,7].

Sulfated fucose-containing polysaccharides of brown algae, fucoidans, exhibit a high antitumor activity, while being non-toxic to normal cells and tissues [8]. Algal fucoidans were found to be effective compounds in combined and adjuvant cancer therapy. Thus, crude fucoidan from *Saccharina japonica* in combination with sorafenib (a VEGFR tyrosine kinase inhibitor) and Avastin^®^ (bevacizumab, an anti-VEGF monoclonal antibody) synergistically inhibited the growth of hepatocellular cells, significantly reduced the expression of the proangiogenic PI3K/AKT/mTOR and KRAS/BRAF/MAPK pathways, and up-regulated the expression level of caspase-3 and -8 proteins [9]. Fucoidans from brown algae *Saccharina cichorioides* and *Fucus evanescens* in combination with polyhydroxysteroids and triterpene glycoside of starfishes possessed strong inhibiting activity on melanoma cell growth [10,11]. Additionally, the efficiency of combined treatment of different types of cancer cells with fucoidans from brown algae and chemotherapeutic drugs such as tamoxifen, paclitaxel, and cisplatin was also reported recently [12,13,14].

Brown algae of the genus *Sargassum* are widely distributed in the World Ocean and have been traditionally used as a food, as well as in in cosmetology and medicine. These algae are a rich source of fucoidans possessing various structures ranging from α-l-fucans with a relatively regular structure to complex polysaccharides, irregular and heterogeneous in their monosaccharide composition [15]. These polysaccharides have valuable biological activities and unique biomedical applications. In particular, it is known that several fucoidans isolated from algae of the genus *Sargassum* have an anticancer effect in various cell lines [16,17,18,19,20,21].

In previous work [22], we isolated fucoidan 2SmF2 from the brown alga *Sargassum muticum* and investigated its structural characteristics and anticancer properties. This galactofucan was shown to inhibit the colony formation of the human colon adenocarcinoma DLD-1 cell line. However, the structural characteristics of 2SmF2 were not established [22].

In this study, we aimed to investigate the structure of fucoidan 2SmF2 from the brown alga *S. muticum* in detail as well as its metabolically oriented effect in combination with 2-DG on colony formation of human melanoma SK-MEL-28 and breast cancer MDA-MB-231 cells.

## 2. Results and Discussion

### 2.1. The Investigation of the Structure of Fucoidan 2SmF2 from S. muticum by Methylation Analysis and NMR Spectroscopy of Its Desulfated, Deacetylated Derivative

The highly purified fucoidan fraction 2SmF2 was obtained from the brown alga *S. muticum* as described previously [22]. It was shown that this polysaccharide was sulfated (23%) and acetylated galactofucan (Fuc:Gal = 3:1). Although the absolute configurations of monosaccharides in these polysaccharides were not determined, we speculate that these polysaccharides consist of l-fucose and d-galactose as has been shown for other known galactofucans from brown algae [23,24].

The desulfation and deacetylation of 2SmF2 were performed to obtain desulfated and deacetylated fucoidan 2SmF2DADS with a yield of 20% of the native fucoidan 2SmF2 sample weight. The obtained derivative was methylated with methyl iodide in the presence of sodium hydroxide in DMSO. The methylated polysaccharide was hydrolyzed, and the resulting mixture of partially methylated monosaccharides was analyzed as alditol acetates by gas–liquid chromatography–mass spectrometry (GLC-MS) [25] (Table 1).

It was shown that fucoidan 2SmF2DADS contained fucose residues linked mainly by 1,4- and 1,3- and, to a lesser extent, by 1,3,4- and 1,2,4-O-glycosidic bonds. Galactose residues were substituted predominantly at position 4, and a small number of 1,3-, 1,6-, and 1,2,6-, 1,3,6-, and 1,4,6-linked galactose residues were also identified. Both fucose and galactose residues were found on the non-reducing ends. Therefore, according to methylation data, this galactofucan had a complex branched structure.

Then, the desulfated and deacetylated fucoidan 2SmF2DADS was investigated by 1D and 2D NMR spectroscopy. 

The ^1^H NMR spectrum contained two main signals in the anomeric region at 4.92–5.29 ppm, signals of fucose methyl groups at 1.3–1.34 ppm, and signals of other protons of the monosaccharide ring at 3.68–4.75 ppm.

The ^13^C NMR spectrum contained signals in the anomeric region at 101.5–102.1 ppm, signals of fucose methyl groups at 16.4–17.2 ppm, signal of the CH_2_OH group (unsubstituted C6 of galactose) at 62.4 ppm, and signals of other carbons (C2–C5) at 68.1–81.6 ppm. 

**Figure 1:**
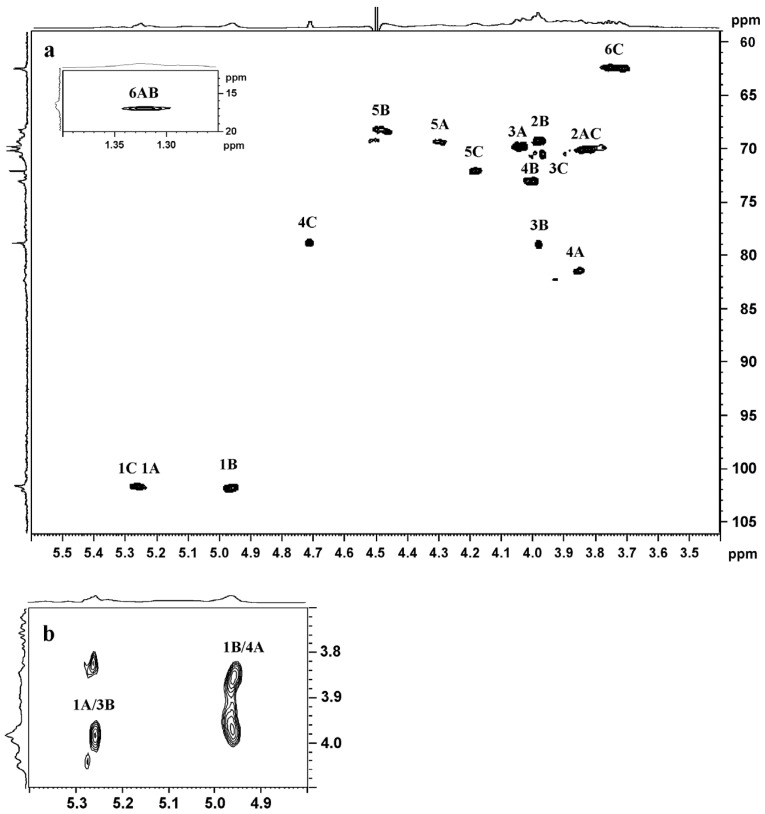
The HSQC (**a**) and a fragment of ROESY (**b**) spectra of deacetylated and desulfated fucoidan 2SmF2DADS.

The 2D NMR spectra revealed the presence of two main types of α-l-fucopyranose residues, A and B, and one type of α-d-galactopyranose residue, C. We identified the signals of their carbons and protons by COSY and HSQC (Figure 1a) NMR spectra. Data are shown in Table 2. 

The downfield shifts of signals of H4/C4 of fucose residue A and galactose residue C at 81.4/3.85 and 78.8/4.71, respectively, as well as H3/C3 of residue B at 79/3.98 ppm suggested that fucose residues were substituted at positions 3 and 4 and galactose residues were substituted at C4. These data are in good agreement with the results of methylation analysis. The types of linkages between fucose residues in the investigated polysaccharide were confirmed by the ROESY spectrum (Figure 1b). The cross-peaks of H1 (A) with H3 (B) and H1 (B) with H4 (A) indicated the presence of 1,3- (A→B) and 1,4- (B→A) bonds in the fucoidan molecules. Thus, 2SmF2DADS contained a main chain of predominantly alternating residues of 1,3- and 1,4-linked α-l-fucopyranose. The way the galactose residues were attached could not be determined. In addition, the chemical shift of H4 of the residue C (at 4.71 ppm) was rather a characteristic signal of its sulfation than of glycosylation. Therefore, the galactose residues could contain sulfate groups, which were not completely removed by the procedure of desulfation.

It is known that galactofucans obtained from algae of the genus *Sargassum* have great structural diversity. Thus, the fucoidan of *S. oligocystum* had a main chain of 1,3-linked α-l-fucopyranose residues and a polysaccharide from *S. mcclurei* contained a 1,3-linked fucose backbone with 1,4-linked inclusions [16,17]. Galactofucan from *S. polycystum* had a main chain of 1,3-linked 4-sulfated fucose residues with single inclusions of 1,2-linked α-d-galactopyranose residues [24]. Fucoidans from *S. duplicatum* and *S. feldmannii* contained 1,4- and 1,3-linked chains, respectively, of α-l-fucopyranose and β-d-galactopyranose residues [26,27]. Sulfated galactofucan with a backbone of a block structure was obtained from *S. thunbergii* [28]. The branches of the above-mentioned galactofucans also were reported to be structurally different.

### 2.2. Mass Spectrometry of the Partial Degradation Products

To confirm the data obtained by the NMR technique and/or find structural peculiarities, invisible by NMR and chemical methods, we have applied a well-established method of partial degradation of the crude polysaccharide in heavy-oxygen water (H_2_^18^O) by autohydrolysis and mass spectrometry techniques for the analysis. Autohydrolysis is denoted here as mild acid hydrolysis where sulfate groups of the polysaccharide act as the source of acid. The incorporation of the ^18^O in the OH-group at the reducing end during autohydrolysis allows labeling of the fragment ions for better identification by mass spectrometer due to the +2 mass shifts.

#### 2.2.1. Mass Spectrometry of Oligosaccharides, Obtained by Autohydrolysis in Heavy-Oxygen Water

**Figure 2:**
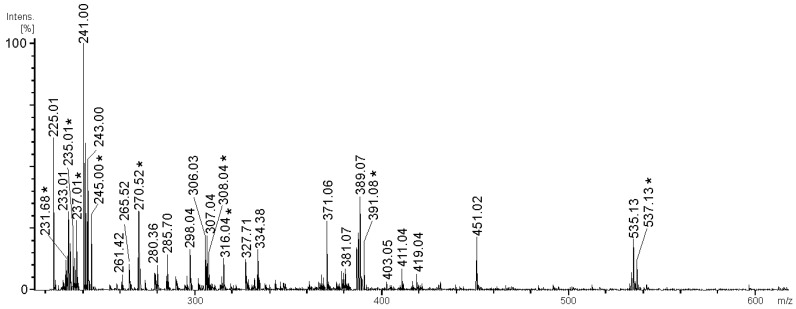
Negative-ion ESIMS of the 2SmF2AH fraction, obtained from 2SmF2 fucoidan sample by autohydrolysis in heavy-oxygen water. ^18^O-labeled ions are marked with an asterisk.

The negative-ion ESIMS of the 2SmF2AH preparation (Figure 2) have shown that the products of autohydrolysis were mostly represented by fucose-containing (degree of polymerization (DP) = 1–4) ions with up to four sulfate groups per ion.

However, some galactose-containing ions were detected (Table 3) as single galactose inserts. We were unable to observe long chains, built up with galactose blocks as was observed earlier for other algae [29,30]. Furthermore, the outcome of a mild acid hydrolysis in TFA (2 h, 80 °C) was similar to the autohydrolysis results reported above. Hence, we speculated that galactose residues do not form blocks with high DP but are rather presented as single branches and terminal galactose residues.

To establish structural features of some fragments found in the mixture 2SmF2AH that could be compared to NMR and GC-MS data, we have recorded MS/MS spectra of the most abundant ions, labeled with ^18^O.

#### 2.2.2. Mass Spectrometry of Oligosaccharides, Obtained by Mild Acid Hydrolysis in Heavy-Oxygen Water

Negative-ion tandem ESIMS of the monosulfated fucose ion at *m/z* 245.00 (Figure S1) have revealed that fucose residues were sulfated mostly in position 2 and less in position 4 of the fucose residue due to the relative intensities of the fragment ions ^0,2^X at *m/z* 140.97 and ^0,2^A at *m/z* 183.00 [31], ^2,4^A at *m/z* 140.97 [32]. A similar pattern of the fragment ion intensities was observed in the autohydrolysis sample from *Fucus evanescens* with a main chain of alternating 1,3- and 1,4-linked fucose residues [32].

MS/MS spectra of the monosulfated [Fuc_2_(SO_3_Na) − Na]^−^ at *m/z* 391.08 and disulfated [Fuc_2_(SO_3_Na)_2_ − 2Na]^2−^ at *m/z* 235.01 fucobiose ions (Figures S2 and S3) were similar to that of the *F. evanescens* samples, obtained in the same conditions [32], suggesting at least three structural variants: Fuc-4-OSO_3_^−^-(1→4)-Fuc, Fuc-(1→2)-Fuc-2(4)-OSO_3_^−^, and Fuc-2(4)-OSO_3_^−^-(1→4)-Fuc-2-OSO_3_^−^. The sample from *F. evanescens*, showed, however, the presence of the doubly charged ^2,4^A_2_-type fragment ion at *m*/*z* 182.00, probably from the structural variant Fuc-2(4)-OSO_3_^−^-(1→4)-Fuc-3-OSO_3_^−^, while the studied disaccharides did not reveal this signal. Possibly, a sulfate group was located in position 3, which was found in fucoidan from *F. evanescens* earlier [33].

MS/MS of the monosulfated [Fuc_3_(SO_3_Na) − Na]^−^ at *m/z* 537.14 ion (Figure S4) had low amount of signals and was interpreted as Fuc-2(4)-OSO_3_^–^-(1→3)-Fuc-(1→2)-Fuc. The presence of 1,2-type linkage was suggested by the ^0,2^X_0_-type ion at *m/z* 433.09 that was distinguished from incorrect interpretation [34] by ^18^O-labeling [32]. Since there were no A-type signals, corresponding to 1,4-type linkages, non-reducing fucose residue was linked by 1,3-type linkages. It is possible that the studied fragment was a branch, represented by a disaccharide unit, which was attached to position 2 of the main chain’s fucose residue.

Tandem negative-ion ESIMS of di- and trisulfated fucotriose ions [Fuc_3_(SO_3_Na)_2_ − 2Na]^2−^ at *m/z* 308.04 and [Fuc_3_(SO_3_Na)_3_ − 3Na]^3−^ at *m/z* 231.68, respectively (Figures S5 and S6), revealed that trisaccharides were composed of a Fuc-(1→4)-Fuc-(1→4)-Fuc backbone, while sulfate groups occupied mostly positions 2 in all fucose residues but the non-reducing fucose, which could be disulfated in positions 2 and 4. The high intensity of ^2,4^A-type ions from cross-ring cleavages could indicate sulfation in position 3 of fucose residues, however, we did not observe any evidence of ^0,3^X/^0,3^A-type ions as co-indicators [31].

**Figure 3:**
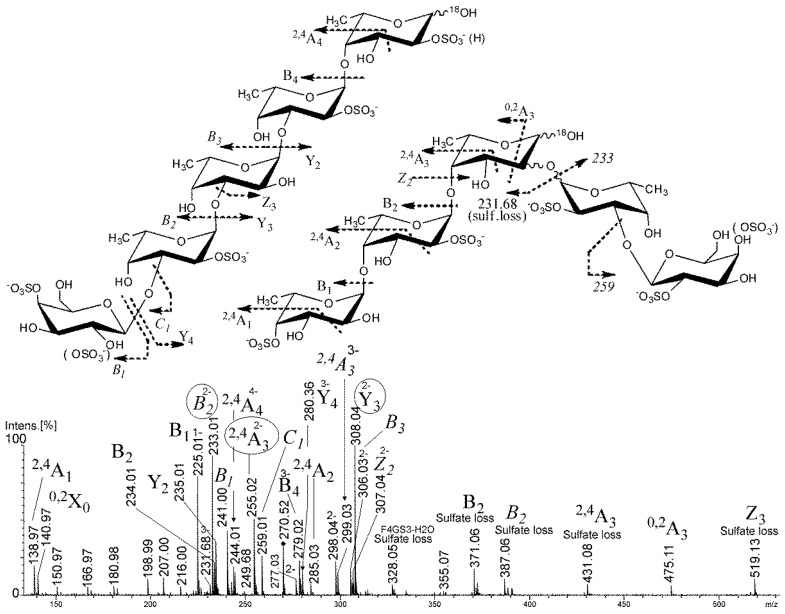
Negative-ion ESIMS/MS of the [Fuc_3_Gal(SO_3_Na)_4_ − 3Na]^4−^ at *m/z* 270.52, labeled with ^18^O.

The galactose-containing components of the 2SmF2AH sample were also examined by negative-ion tandem ESIMS. The ion [Fuc_2_Gal(SO_3_Na)_3_ − 3Na]^3−^ at *m/z* 237.01 (Figure S7) was found to have two structural variants: Gal-4(2)-OSO_3_^−^-(1→3)-Fuc-4-OSO_3_^−^-(1→4)-Fuc-2-OSO_3_^−^ and branched Gal-4(2)-OSO_3_^−^-(1→3)-Fuc-2-O-Fuc-2(4)-OSO_3_^−^. Galactose residues were found to be terminal and linked with 3-type linkages that are common to sulfated galactofucans from algae of the orders Laminariales [29,30] and Fucales [17,35]. The most complex negative-ion ESIMS of a tetrasulfated tetrasaccharide [Fuc_4_Gal(SO_3_Na)_4_ − 4Na]^4−^ ion at *m/z* 270.52 was also found in branched and linear variants (Figure 3). The mass spectrum showed the three most significant fragment ions (Figure 3, encircled) from glycosidic bond cleavage at *m/z* 233.01 of B type and 308.04 of Y type which suggested the structural variant of the ion under study to be Gal-(1→3)-Fuc-(1→3)-Fuc-(1→3)-Fuc-(1→4)-Fuc due to the absence of ^2,4^A-type ions [32] from the cross-ring cleavages (Figure 3, leftmost structure). The sulfate group positions were assigned to position 2 due to the intensities of the corresponding B-type ions in the non-reducing/internal saccharide residues and in position 4 due to the presence of the ^2,4^A_1_-type ion of the non-reducing residues of the saccharides. The presence of the ^0,2^A-type ion of the fucose residue at the reducing end indicated the unsubstituted OH-group in positions 3 [31] and thus 1,4-type linkage. ^0,2^X type at *m/z* 140.97 indicated sulfation at position 2 of the fucose residue at the reducing end. Thus, the linear structural variant of the selected ion (Figure 3, leftmost structure) was Gal-4(2)-OSO_3_^−^-(1→3)-Fuc-2-OSO_3_^−^-(1→3)-Fuc-2-OSO_3_^−^-(1→3)-Fuc-2-OSO_3_^−^-(1→4)-Fuc-2-OSO_3_^−^.

Due to the presence of both ^0,2^A_3_-type ion at *m/z* 277.03 and ^2,4^A_3_-type fragment ion at *m/z* 255.02 from cross-ring cleavage of fucose residue at the reducing end and releasing a trisaccharide ion without galactose residue, it was concluded that another branched structural variant of the selected ion was present (Figure 3, rightmost structure).

Due to ^18^O-labeling, it was possible to distinguish between previously incorrectly assigned ^0,2^X-type fragment ions [34] which, further, were correctly shown to be ^2,4^A-type ions from the cross-ring cleavage of 1,4-linked saccharide residues [32]. Thus, the presence of ^2,4^A-type series of fragment ions at *m/z* 138.97 (indicating substitution by sulfate in positions 4 of the non-reducing fucose residue), *m/z* 285.03 (low intensity, so 1,3-type linkage could not be excluded), and *m/z* 255.02 together with ^0,2^A_3_ at *m/z* 277.03 suggested that the mixture contained two variants of the fucose-containing trisaccharide core: Fuc-4(2)-OSO_3_^−^-(1→4)-Fuc-2-OSO_3_^−^-(1→4)-Fuc and, possibly, Fuc-4(2)-OSO_3_^−^-(1→3)-Fuc-2-OSO_3_^−^-(1→4)-Fuc. Since there were no X-type ions found (like in 1,2-linked fucose residues) to unambiguously detect branching in position 2 of the reducing fucose residue, it was possible to find Z_2_-type fragment ion at *m/z* 307.04 from the glycosidic bond cleavage of the fucose residue at the reducing end, carrying a Fuc-Gal disaccharide unit. In addition, a counter-ion at *m/z* 231.68 indicated a loss of a trisulfated fucotriose and one sulfate group. All this suggested that the studied structural variant had a branched structure, consisting of doubly sulfated fucotriose and a doubly-sulfated Gal-(1→3)-Fuc disaccharide, attached at position 2 of the fucotriose at the reducing end.

The further investigation of the fucoidan fraction 2SmF2AH partly degraded by autohydrolysis in H_2_^18^O by negative-ion tandem ESI mass spectrometry has revealed that the oligosaccharide mixture contained a set of monosulfated and multiply sulfated (up to four) fucooligosaccharides (DP = 1–4) with single insertions of galactose residues at non-reducing ends, which were 1,3-linked to fucose residues of oligosaccharides or resided similarly in short branches, represented by Gal-(1→3)-Fuc disaccharides. Sulfate groups occupied positions 2 and/or 4 of the oligosaccharides. No sulfation at position 3 was detected in ESIMS/MS of monosaccharides in the 2SmF2AH mixture. It was unambiguously confirmed that some oligosaccharides had an alternating 1,3-, 1,4-type linkage, while others had monotonous 1,3-type and 1,4-type linkages (DP up to 3). Branching was found in position 2 of the reducing end, which was represented by single fucose residues or the above-mentioned Gal-(1→3)-Fuc disaccharides.

### 2.3. The Investigation of the Metabolically Oriented Effect of Fucoidan 2SmF2 from S. muticum

The prevalence of aerobic glycolysis is considered to be one of the main metabolic changes that occur in cancer cells. Therefore, most cancer cells consume a higher amount of glucose to generate energy and support metabolic function. These cells are also more dependent on aerobic glycolytic metabolism to generate ATP than on mitochondrial oxidative phosphorylation [36,37]. The resistance of cancer cells to chemotherapeutic agents and radiation therapy which occur under hypoxic conditions due to the activation of glycolysis represents an important challenge in cancer treatment [38].

Glucose analogue 2-DG is used to inhibit glycolysis by mimicking the glucose substrate [39]. 2-DG is actively taken up by cancer cells via hexose transporters and further phosphorylated to 2-deoxy-d-glucose-6-phosphate (2-DG6P) but cannot be completely metabolized. Carbohydrate metabolism is consequently impaired by the inhibition of glycolytic enzymes as a result of the accumulation of 2-DG6P [40,41]. That is why 2-DG has been considered as a possible anticancer agent.

**Figure 4:**
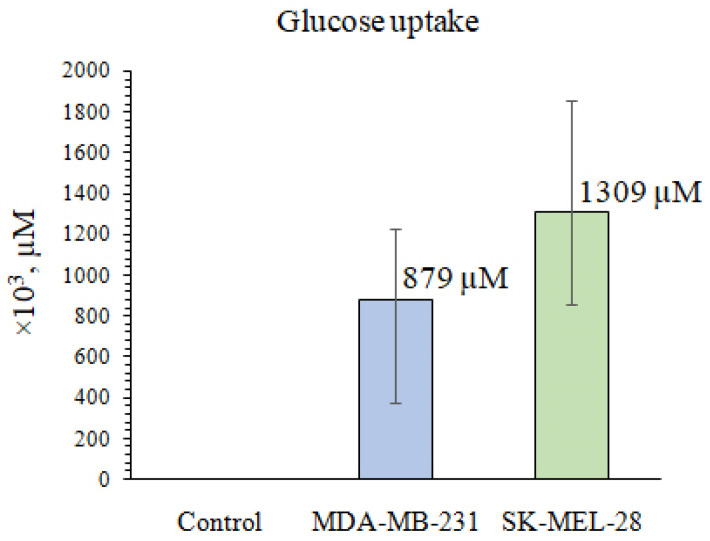
The ability of MDA-MB-231 or SK-MEL-28 cells to take up 2-DG as determined by Glucose Uptake Colorimetric Assay. “Control” is a reaction in which no 2-DG was added, so 2-DG6P was not produced. Data show the mean of three independent experiments ± SD.

In the present study, we measured glucose uptake by melanoma SK-MEL-28 and breast cancer MDA-MB-231 cells using a method based on the detection of 2-DG6P content. It has been shown that SK-MEL-28 and MDA-MB-231 cells have a high level of glucose uptake and a high rate of aerobic glycolysis, which is characteristic of cells with a glycolytic bioenergetic phenotype (Figure 4).

To investigate the combined metabolically oriented effect of 2SmF2 with 2-DG, first of all, the cytotoxicity of investigated compounds was determined.

**Figure 5:**
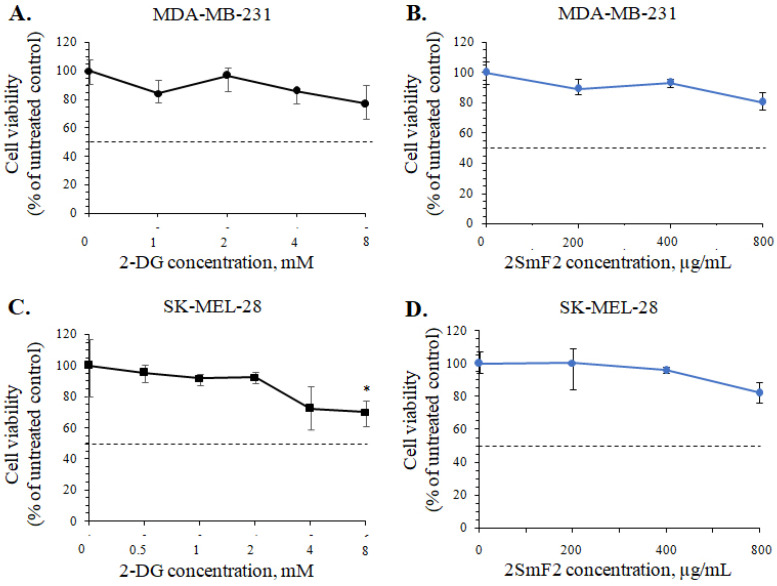
The cytotoxic effects of 2-DG and fucoidan 2SmF2 against breast cancer MDA-MB-231 and melanoma SK-MEL-28 cells.MDA-MB-231 and SK-MEL-28 cells were treated by (**A**,**C**) 2-DG (0.5–8 mM) and (**B**,**D**) 2SmF2 (200–800 µg/mL) and incubated for 24 h. The cell viability was estimated by MTS assay and by the counting of cells in a hemocytometer. The data are presented as mean ± SD for triplicate experiments. A one-way ANOVA and Tukey’s HSD test for multiple comparisons indicated the statistical significance (* *p*  <  0.05).

2-DG exhibited a minor cytotoxic activity against MDA-MB-231 and SK-MEL-28 cells within 24 h at concentrations up to 8 mM (Figure 5A,C), while 2SmF2 fucoidan showed no cytotoxic effect in the range of concentrations from 200 to 800 µg/mL (Figure 5B,D). Doxorubicin was used as a positive control, IC_50_ was 27 µM for SK-MEL-28 cells and 18 µM for MDA-MB-231 cells (Figure S8). Recently, fucoidans from brown algae of the genus *Sargassum* were found to differently affect the viability of cancer cells. Thus, fucoidan extracted from *Sargassum plagiophyllum* (27.82 mg/mL) by microwave-assisted extraction possessed cytotoxic activity against HeLa cervical cancer cells [18]. Ji Y.-B. et al. reported that fucoidan from *S. fusiforme* (SFPS-B2) inhibited the growth of the human gastric cancer cell line SGC-7901 with IC_50_ of 189.30 μg/mL by the induction of apoptosis via a mitochondrial-mediated pathway [19]. Fucoidan F2 from the brown alga *S. polycystum* exhibited a cytotoxic effect against MCF-7 breast cancer cells and HCT-15 colorectal adenocarcinoma cells with IC_50_ of 20 and 50 μg/mL, respectively [20]. On the other hand, sulfated polysaccharide isolated from *S. hemiphyllum* did not exhibit cytotoxicity against 5637 and T24 bladder cancer cells but suppressed cancer stem cell formation which is implicated in tumor initiation, metastatic spread, drug resistance, and tumor recurrence [21]. Also, fucoidans from brown algae *S. oligocystum*, *S. duplicatum*, and *S. feldmannii* were determined to be less cytotoxic against human melanoma SK-MEL-28, colon HT-29, and breast MDA-MB-231 cancer cells at concentrations up to 400 μg/mL [16].

**Figure 6:**
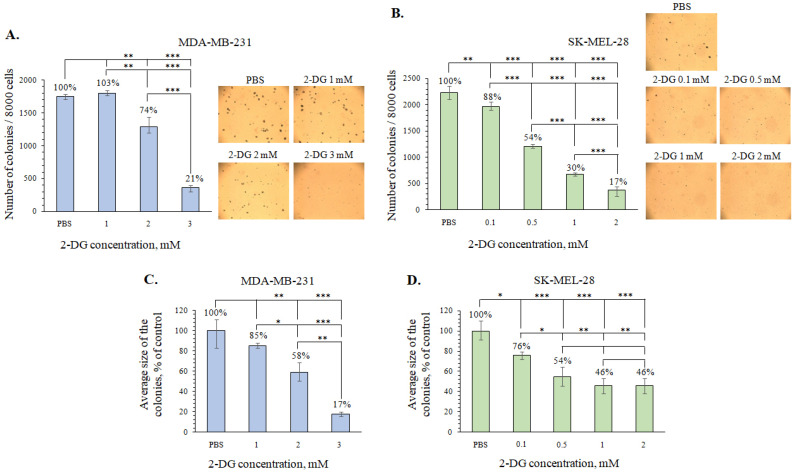
Inhibitory effects of 2-DG on colony formation in MDA-MB-231 and SK-MEL-28 cells. (**A**,**C**) MDA-MB-231 cells were treated with 2-DG at concentrations of 1, 2, and 3 mM and (**B**,**D**) SK-MEL-28 cells were treated with 2-DG at 0.1, 0.5, 1, and 2 mM in soft agar. The number (**A**,**B**) and size (**C**,**D**) of colonies were determined using a microscope (at a total magnification of 40×) and ImageJ software version 1.50i bundled with 64-bit Java 1.6.0_24 (“NIH”, Bethesda, MD, USA). Results are presented as mean ± standard deviation (SD). A one-way ANOVA and Tukey’s HSD test for multiple comparisons indicated the statistical significance (* *p*  <  0.05, ** *p*  <  0.01, *** *p*  <  0.001).

Based on cytotoxicity data, low non-toxic concentrations of 2-DG and 2SmF2 at which they alone did not influence cell viability significantly were chosen to be further tested in the colony formation assay. It was determined that 2-DG at 1, 2, and 3 mM decreased the number of the colonies of MDA-MB-231 cells by 0%, 26%, and 79%, respectively (Figure 6A), and size of colonies by 15%, 42%, and 83%, respectively (Figure 6C). The effect of 2-DG at the concentration of 0.1, 0.5, 1, and 2 mM on colony formation of SK-MEL-28 cells was more pronounced and decreased the number of colonies by 12%, 46%, 70%, and 83%, respectively (Figure 6B). 2-DG at 0.1, 0.5, 1, and 2 mM was shown to reduce colony size of SK-MEL-28 cells by 24%, 46%, 54%, and 54%, respectively (Figure 6D). 

**Figure 7:**
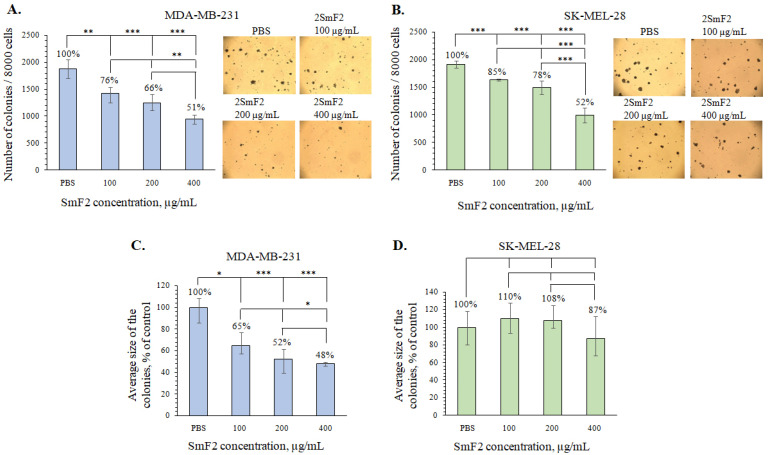
Inhibitory effect of 2SmF2 on the colony formation in MDA-MB-231 and SK-MEL-28 cells. (**A**,**C**) MDA-MB-231 and (**B**,**D**) SK-MEL-28 cells were treated with 2SmF2 at concentrations of 100, 200, and 400 µg/mL in soft agar. The number (**A**,**B**) and size (**C**,**D**) of colonies were counted using a microscope (at a total magnification of 40×) and ImageJ software version 1.50i bundled with 64-bit Java 1.6.0_24 (“NIH”, Bethesda, MD, USA). Results are presented as mean ± standard deviation (SD). A one-way ANOVA and Tukey’s HSD test for multiple comparisons indicated the statistical significance (* *p*  <  0.05, ** *p*  <  0.01, *** *p*  <  0.001).

2SmF2 alone was demonstrated to possess slight inhibiting activity on the formation and growth of cancer cell colonies in soft agar. The fucoidan 2SmF2 at concentrations of 100, 200, and 400 µg/mL decreased the number of colonies of MDA-MB-231 cells by 24, 34, and 49%, respectively (Figure 7A), and SK-MEL-28 cells by 15, 22, and 48%, respectively (Figure 7B). 2SmF2 at 100, 200, and 400 µg/mL was found to reduce the size of colonies of MDA-MB-23 cells by 35%, 48%, and 52%, respectively (Figure 7C), while those of SK-MEL-28 by 0%, 0%, and 13%, respectively (Figure 7D).

These results were in accordance with previously published data [16,26,27]. Thus, recently we showed that galactofucans from brown algae *S. duplicatum* and *S. feldmannii* (200 μg/mL) slightly suppressed colony formation in SK-MEL-28 melanoma cells and MDA-MB-231 breast adenocarcinoma cells but significantly decreased the number of colonies of DLD-1 colon adenocarcinoma cells. Also, Thinh et al. reported that three fucoidan fractions extracted from *S. mcclurei* were less cytotoxic and exhibited an inhibiting effect on the colony formation in colon cancer DLD-1 cells [17].

**Figure 8:**
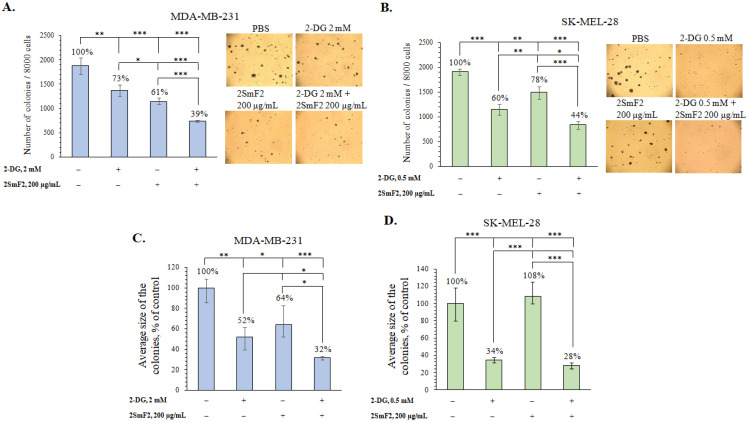
Inhibitory effects of 2SmF2 in combination with 2-DG on colony formation of MDA-MB-231 and SK-MEL-28 cells. (**A**,**C**) MDA-MB-231 and (**B**,**D**) SK-MEL-28 cells were treated with 2SmF2 (200 µg/mL) in combination with 2-DG (2 or 0.5 mM) in soft agar. The number (**A**,**B**) and size (**C**,**D**) of colonies were determined using a microscope (at a total magnification of 40×) and ImageJ software. Results are presented as mean ± standard deviation (SD). A one-way ANOVA and Tukey’s HSD test for multiple comparisons indicated the statistical significance (* *p*  <  0.05, ** *p*  <  0.01, *** *p*  <  0.001).

Next, the combined effect of 2SmF2 fucoidan and 2-DG—a competitive inhibitor of glycolysis—was tested on the model of colony formation using MDA-MB-231 and SK-MEL-28 cells. We hypothesized that the fucoidans could potentiate the inhibiting effect of low concentrations of 2-DG and effectively decrease the number of colonies of tested cancer cells. Thus, the combination of 2-DG (2 mM) with 2SmF2 (200 µg/mL) inhibited the MDA-MB-231 cell colony formation by 61% compared to non-treated cells (control) (Figure 8A). The treatment of SK-MEL-28 cells with a combination of 2-DG (0.5 mM) and 2SmF2 (200 µg/mL) led to reduction of the number of colonies by 56% compared to control (Figure 8B). It is worth noting that the combination of fucoidan with 2-DG significantly reduced the size of colonies of MDA-MB-231 and SK-MEL-28 cells (Figure 8C,D).

The metabolically oriented effects of algal polysaccharides have been poorly investigated. It was reported that fucoidan from the brown alga *Undaria pinnatifida* possessed antidiabetic activity via stimulation of glucose uptake in normal 3T3 adipocytes and restored the insulin-stimulated glucose uptake in obesity-induced insulin-resistant adipocytes [42]. Fucoidan from the brown alga *Ascophyllum nodosum* was found to regulate blood glucose levels by direct inhibition of glucose transport via SGLT1, causing the glucose transport to markedly reduce and relieve postprandial hyperglycemia [43]. Fucoidan from *Saccharina cichorioides* in combination with 2-DG was capable of significant inhibition of the viability, proliferation, and colony formation of SK-MEL-28 human melanoma cells [44]. The results of this work provide evidence that fucoidan from the brown alga *S. muticum* potentiates the inhibitory effect of 2-DG and significantly suppressed the progression of human melanoma SK-MEL-28 and breast adenocarcinoma MDA-MB-231 cells.

## 3. Materials and Methods

### 3.1. Materials

Commercially available organic solvents, inorganic acids and salts, sodium hydroxide, trichloroacetic acid (TCA), and trifluoroacetic acid (TFA) were used (Laverna-Lab, Moscow, Russia). Alcian blue was obtained from PanReac AppliChem (Barcelona, Spain) and H_2_^18^O from Component-Reaktiv (Moscow, Russia). Standards (mannose, rhamnose, glucose, galactose, and xylose), O-toluidine blue, phosphate-buffered saline (PBS), L-glutamine, penicillin–streptomycin solution (10,000 U/mL, 10 μg/mL), trypsin, basal medium Eagle (BME), and Dulbecco’s modified Eagle’s medium (DMEM) were purchased from Sigma-Aldrich (St. Louis, MO, USA). The sorbent for chromatography, Amberlite CG-120, was obtained from Serva Electrophoresis GmbH (Heidelberg, Germany) and Sep-Pak C18 cartridge from Waters Corporation (Milford, MA, USA). MTS reagent—3-[4,5-dimethylthiazol-2-yl]-2,5-diphenyltetrazolium bromide—was purchased from Promega Corporation (Madison, WI, USA) and doxorubicin (Doxo) from Teva Pharmaceutical Industries, Ltd. (Tel Aviv, Israel). The fetal bovine serum (FBS) was obtained from Biowest (Bradenton, FL, USA). 2-Deoxy-D-glucose was purchased from Alfa Aesar (Miaoli, Taiwan, China).

Samples of the alga *Sargassum muticum* were collected in June 2015 on the coast of the Yellow Sea (People’s Republic of China). Fresh algal biomass was powdered and pretreated with 70% aqueous ethanol (*w*/*v* = 1:10) for 10 days. The defatted algal biomass was air-dried at room temperature.

### 3.2. Instruments

Nuclear magnetic resonance (NMR) spectra (1D and 2D experiments) were obtained using an Avance DPX-500 NMR spectrometer (Bruker BioSpin Corporation, Billerica, MA, USA) at 35 °C. The concentration of the samples was 15 mg of polysaccharide/mL of D_2_O.

GLC-MS of alditol acetate derivatives was performed using a Hewlett-Packard 6850 (Agilent Technologies, Santa Clara, CA, USA) chromatograph equipped with HP-5MS capillary column (30 m × 0.2 mm) with a temperature gradient of 150 → 230 °C at 3 °C min^−1^.

Electrospray ionization mass spectra (ESIMS) were recorded with an Impact II Q-TOF mass spectrometer (Bruker BioSpin Corporation, Billerica, MA, USA). All spectra were acquired in the negative-ion mode, precalibrated with a standard “HP-mix” (Agilent Technologies, Santa Clara, CA, USA) for negative-ion mode at default instrument settings. The samples were diluted with ACN:H_2_O (1:1) to approx. 0.01 mg/mL and introduced into the mass spectrometer at a flow rate of 5 µL/min using a syringe pump (KD Scientific, Holliston, MA, USA). The mass spectrometry detection was performed using a CaptiveSpray (Bruker Daltonics, Bremen, Germany) ionization source at a capillary voltage of 1.3 kV. Collision-induced dissociation (CID) product ion mass spectra were recorded in auto-MS/MS mode with a collision energy of 43 eV. The precursor ions were isolated with an isolation width of 1 mass unit.

### 3.3. General Methods

#### 3.3.1. Isolation of Fucoidan 2SmF2 from *S. muticum*

Galactofucan 2SmF2 from *S. muticum* was obtained as described in the previous work [22]. In brief, a sample of defatted, dried, and powdered algal frond (200 g) was extracted twice with 0.1 M HCl (2 L) for 2 h at 60 °C. The extracts were combined, neutralized, dialyzed against distilled water, concentrated under a vacuum, and lyophilized. The obtained water-soluble polysaccharide fraction was dissolved in 0.04 M HCl and centrifuged. The supernatant was applied to a DEAE-cellulose column (Cl^−^ form, 7 × 20 cm) equilibrated with 0.1 M NaCl. The laminaran-containing fraction was eluted with water. Then, the column was successively eluted with a linear gradient of NaCl (from 0.1 to 2 M). The obtained eluates were analyzed by the phenol–sulfuric acid method [45]. The obtained fractions of fucoidans (F) 2SmF1 and 2SmF2 were concentrated under a vacuum, dialyzed, and lyophilized.

#### 3.3.2. Modification of Fucoidan 2SmF2

The fucoidan SmF2 (100 mg) was deacetylated and desulfated as previously de-scribed [22]. The yield of deacetylated (DA) and desulfated (DS) derivative 2SmF2DADS was 18 mg.

#### 3.3.3. Methylation of Desulfated and Deacetylated Fucoidan 2SmF2DADS

The desulfated and deacetylated fucoidan 2SmF2DADS was methylated using a modification of the previously described NaOH slurry method [46]. In brief, a sample of the fucoidan 2SmF2DADS (2 mg) was solubilized in DMSO (1 mL), and powdered NaOH (100 mg) was added to the solution, followed by MeI (0.2 mL). The mixture was stirred for 20 min, and NaOH and MeI were added again. This mixture was stirred periodically for 1 h, and then it was cooled on ice. The reaction was terminated by the addition of 1 mL of water. The excess MeI was removed by concentration under a vacuum, and the resulting solution was passed through a Sep-Pak C18 cartridge. Methylated fucoidan was eluted with 50% MeOH, concentrated under a vacuum, and hydrolyzed with 2 M TFA at 100 °C for 6 h. TFA was neutralized with aqueous NH_3_ (5%), and the resulting solution was concentrated and lyophilized under a vacuum. Then, the resulting monosaccharides were reduced with NaBH_4_ and acetylated with Ac_2_O in pyridine. Partially methylated alditol acetates were analyzed by GLC-MS.

#### 3.3.4. Autohydrolysis of Fucoidan 2SmF2

The sample of fucoidan 2SmF2 (2.5 mg) was dissolved in 50 μL of H_2_^18^O. Then, the fucoidan was converted to the H^+^-form using an Amberlite CG-120 cation-exchange mini-column (200–400 mesh) with 0.5 mL of H_2_^18^O and left at 37 °C for 72 h. The mixture was neutralized with NH_4_HCO_3_ and lyophilized to obtain fraction 2SmF2AH.

#### 3.3.5. Mild Acid Hydrolysis of Desulfated and Deacetylated Fucoidan 2SmF2DADS

The sample of 2SmF2DADS (1 mg) was dissolved in 175 μL of H_2_^18^O with the addition of 25 μL of 4 M TCA. Then, the obtained solution was heated to 80 °C for 2 h. The mixture was neutralized with NH_4_HCO_3_ and lyophilized.

### 3.4. Anticancer Activity

#### 3.4.1. Cell Culturing

SK-MEL-28 human melanoma cells (ATCC # HTB-72™) and MDA-MB-231 human breast adenocarcinoma cells (ATCC # HTB-26^TM^) were grown in a monolayer in DMEM, supplemented with 10% (*v*/*v*) heat-inactivated FBS and 1% penicillin–streptomycin. Cell cultivation was carried out in humidified atmosphere containing 5% CO_2_.

#### 3.4.2. Glucose Uptake Assay

MDA-MB-231 and SK-MEL-28 cells (2.5 × 10^4^/mL) were seeded in 96-well plates and cultured at 37 °C in 200 μL of DMEM with 10% FBS in a 5% CO_2_ incubator for 24 h. Then, the medium was removed and cells were washed twice with 100 µL PBS and used to determine the level of glucose uptake using a Glucose Uptake-Glo^TM^ Assay (Promega, Fitchburg, WI, USA) according to the manufacturer’s protocol. The content of 2-DG6P was determined by the luminescence method using a PHERAStar FS luminometer (BMG Labtech, Offenburg, Germany) with appropriate calibration curves.

#### 3.4.3. Cell Cytotoxicity Assay

SK-MEL-28 and MDA-MB-231 cells were seeded (6 × 10^3^/mL) in 96-well plates in 200 μL of DMEM with 10% FBS and incubated for 24 h at 37 °C in a 5% CO_2_ incubator. Then, the medium was removed and replaced by a fresh medium containing: (a) PBS (control); (b) fucoidan 2SmF2 at different concentrations (100, 200, 400, or 800 µg/mL); (c) 2-DG at different concentrations (1, 2, 4, 8 mM for MDA-MB-231 and 0.5, 1, 2, 4 mM for SK-MEL-28 cell lines); (d) doxorubicin at different concentrations (12.5, 25, 50, 100 µM), and incubated for 24 h at 37 °C in a 5% CO_2_ incubator. The culture media were carefully aspirated from the fucoidan-treated cells, and every well was washed with 100 μL of PBS. The cell monolayer was treated with 50 μL of trypsin and incubated for 3 min. The cells were collected with 150 μL of an appropriate complete culture medium and resuspended. The number of cells was counted using a hemocytometer under a Motic AE 20 microscope (XiangAn, Xiamen, China). The number of living cells treated with 2-DG was determined using an MTS assay.

#### 3.4.4. Soft Agar Cologenic Assay

A soft agar assay was performed on the MDA-MB-231 and SK-MEL-28 cell lines with the following scheme. The cells (2.4 × 10^4^/mL) were treated by: (a) PBS (control); (b) 100, 200, or 400 µg/mL of 2SmF2; (c) 0.1–3 mM of 2-DG; (d) 200 µg/mL of 2SmF2 and 2-DG (2 mM for MDA-MB-231 and 0.5 mM for SK-MEL-28 cell lines). Then, the cells were grown in 1 mL of 0.3% agar in basal medium Eagle (BME) containing 10% of FBS. The culture was maintained in the 5% CO_2_ incubator for 2 weeks, at 37 °C, and the cell colonies and their average size were scored using a microscope and ImageJ software [47].

#### 3.4.5. Data Analysis

Every figure shown in this study represents at least three independent experiments with similar results. Statistical differences were evaluated using one-way ANOVA [48] and Tukey’s HSD test with * *p* ≤ 0.05; ** *p* ≤ 0.01; *** *p* ≤ 0.001.

## 4. Conclusions

In conclusion, it was shown that fucoidan 2SmF2 is a sulfated and acetylated branched galactofucan of a complex structure with a main chain of 1,3- and 1,4-linked fucose residues. 1,3-linked and 1,4-linked extended fucose fragments (DP up to 3), disaccharide units of α-d-Gal-(1→3)-α-L-Fuc, and 1,3,4- and 1,2,4-linked fucose branching points were also found in the structure of the polysaccharide. The sulfate groups were at positions 2 and 4 of fucose and galactose residues. The feasibility and efficacy of a combined metabolically oriented effect of natural sulfated polysaccharide from the brown alga *S. muticum* and the glycolysis inhibitor 2-DG to inhibit the aggressive human melanoma SK-MEL-28 cells and breast adenocarcinoma MDA-MB-231 cells were assessed. Further in-depth investigation of the metabolically oriented effects of this algal polysaccharide should further clarify the mechanism of the effect on cancer metabolism and facilitate development of effective adjuvant approach for cancer treatment.

## Figures and Tables

**Table 1:** Methylation analysis of the desulfated, deacetylated fucoidan 2SmF2DADS.

Partially Methylated Fucitol or Galactitol Acetates	mol%	Linkage Type
2,3,4-tri-O-methyl-fucitol	5	Fuc1→
2,3-di-O-methyl-fucitol	26	→4Fuc1→
2,4-di-O-methyl-fucitol	20	→3Fuc1→
2-O-methyl-fucitol	15	→3,4Fuc1→
3-O-methyl-fucitol	11	→2,4Fuc1→
2,3,4,6-tetra-O-methyl-galactitol	3	Gal1→
2,3,6-tri-O-methyl-galactitol	13	→4Gal1→
2,4,6-tri-O-methyl-galactitol	1	→3Gal1→
2,3,4-tri-O-methyl-galactitol	2	→6Gal1→
3,4-di-O-methyl-galactitol	1	→2,6Gal1→
2,3-di-O-methyl-galactitol	2	→4,6Gal1→
2,4-di-O-methyl-galactitol	1	→3,6Gal1→

**Table 2:** NMR data for the desulfated and deacetylated fucoidan 2SmF2DADS.

Residue	C1/H1	C2/H2	C3/H3	C4/H4	C5/H5	C6/H6
A →4)-α-l-Fuc*p*-(1→	101.7/5.25	70.0/3.82	69.8/4.04	81.4/3.85	69.4/4.29	16.9/1.32
B →3)-α-l-Fuc*p*-(1→	101.8/4.97	69.3/3.97	79/3.98	73/4.0	68.4/4.48	16.9/1.32
C →4)-α-d-Gal*p*-(1→ or α-d-Gal*p*-4OSO_3_^−^-(1→	101.7/5.26	70.0/3.82	70.5/3.97	78.8/4.71	72/4.17	62.4/3.77, 3.72

**Table 3:** Composition of the oligosaccharide mixture found by negative-ion ESIMS of 2SmF2AH fraction.

*m*/*z*	Composition	*m*/*z*	Composition
231.01	[Fuc_3_(SO_3_Na)_3_ − 3Na]^3−^	306.03	[Fuc_2_Gal(SO_3_Na)_2_ − 2Na − H_2_O]^2−^
231.68	[Fuc_3_(SO_3_Na)_3_ − 3Na − O + ^18^O]^3−^	308.04	[Fuc_3_(SO_3_Na)_2_ − 2Na − O + ^18^O]^2−^
233.01	[FucGal(SO_3_Na)_2_ − 2Na − H_2_O]^2−^	316.04	[Fuc_2_Gal(SO_3_Na)_2_ − 2Na − O + ^18^O]^2−^
235.01	[Fuc_2_(SO_3_Na)_2_ − 2Na − O + ^18^O]^2−^	327.71	[Fuc_4_Gal(SO_3_Na)_3_ − 3Na − H_2_O]^3−^
237.01	[Fuc_2_Gal(SO_3_Na)_3_ − 3Na − O + ^18^O]^3−^	334.38	[Fuc_4_Gal(SO_3_Na)_3_ − 3Na − O + ^18^O]^3−^
243.00	[FucSO_3_Na − Na)]^−^	389.07	[Fuc_2_SO_3_Na − Na]^−^
245.00	[FucSO_3_Na − Na − O + ^18^O]^−^	391.08	[Fuc_2_SO_3_Na − O + ^18^O]^−^
270.52	[Fuc_4_Gal(SO_3_Na)_4_ − 4Na]^4−^	535.31	[Fuc_3_SO_3_Na − Na]^−^
280.36	[Fuc_4_(SO_3_Na)_3_ − 3Na − O + ^18^O]^3−^	537.13	[Fuc_3_SO_3_Na − Na − O + ^18^O]^−^
285.70	[Fuc_3_Gal(SO_3_Na)_3_ − 3Na]^3−^		

## Data Availability

The data presented in this study are available on request from the corresponding author.

## Supplementary Materials

The following supporting information can be downloaded at: https://www.mdpi.com/article/10.3390/md21090486/s1, Figure S1. Negative-ion ESIMS/MS of the ion [Fuc(SO_3_Na) − Na]^−^ at *m/z* 245.00, labeled with ^18^O; Figure S2. Negative-ion ESIMS/MS of the ion [Fuc_2_(SO_3_Na) − Na]^−^ at *m/z* 391.08, labeled with ^18^O; Figure S3. Negative-ion ESIMS/MS of the ion [Fuc2(SO_3_Na)_2_ − 2Na]^2−^ at *m/z* 235.01, labeled with ^18^O; Figure S4. Negative-ion ESIMS/MS of the ion [Fuc_3_SO_3_Na − Na]^−^ at *m/z* 537.14, labeled with ^18^O; Figure S5. Negative-ion ESIMS/MS of the ion [Fuc_3_(SO_3_Na)_2_ − 2Na]^2−^at *m/z* 308.04, labeled with ^18^O; Figure S6. Negative-ion ESIMS/MS of the ion [Fuc_3_(SO_3_Na)_3_ − 3Na]^3−^ at *m/z* 231.68 (with ^18^O label); Figure S7. Negative-ion ESIMS/MS of the ion [Fuc_2_Gal(SO_3_Na)_3_ − 3Na]^3−^ at *m/z* 237.01, labeled with ^18^O; Figure S8. The cytotoxic effects of doxorubicin against breast cancer MDA-MB-231(**A**) and melanoma SK-MEL-28 (**B**) cells.
