# Supplementary material for: Structure and Metabolically Oriented Efficacy of Fucoidan from Brown Alga Sargassum muticum in the Model of Colony Formation of Melanoma and Breast Cancer Cells
Source: Mar Drugs. 2023 Sep 10;21(9):486. doi: 10.3390/md21090486 (PMC10532595; doi:10.3390/md21090486)
Supplement: Supplementary file 1 [file marinedrugs-21-00486-s001.zip › marinedrugs-2596991-supplementary.pdf]

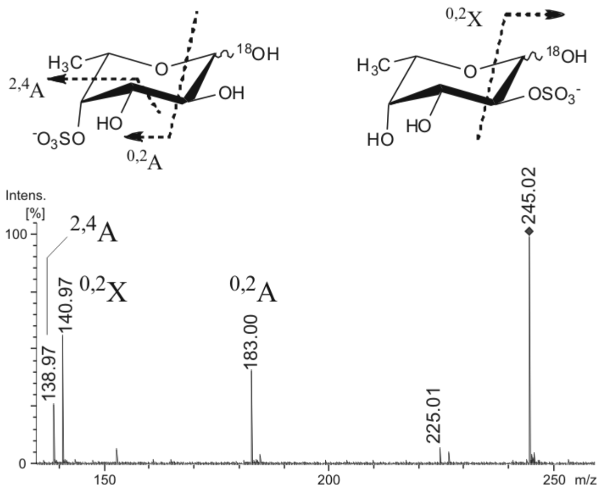

Figure S1. Negative-ion ESIMS/MS of the ion [Fuc(SO₃Na) – Na]⁻ at *m/z* 245.00, labeled with ^{18}O

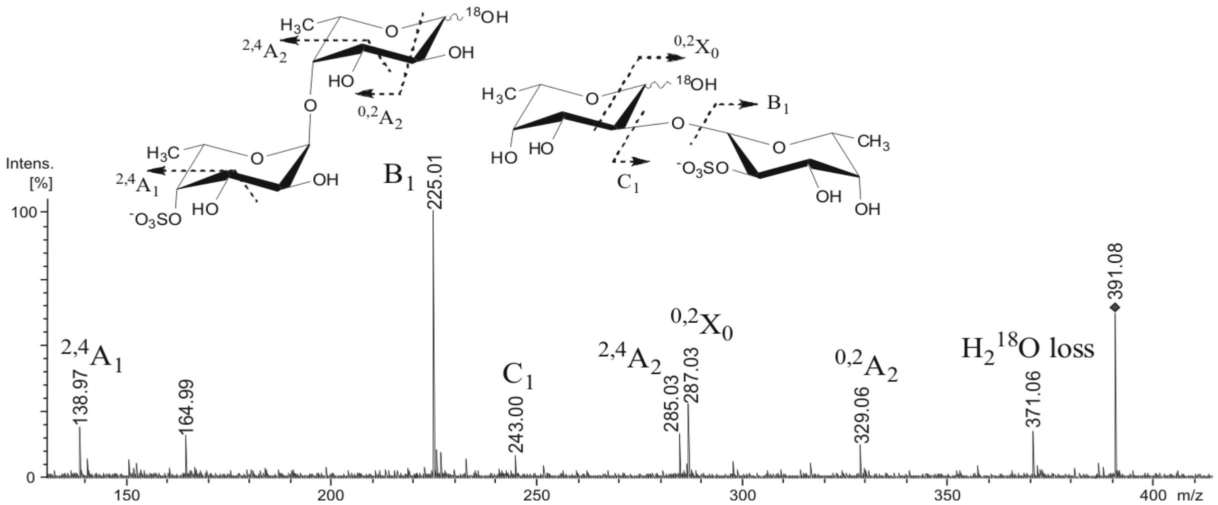

Figure S2. Negative-ion ESIMS/MS of the ion [Fuc₂(SO₃Na) – Na]⁻ at *m/z* 391.08, labeled with ^{18}O

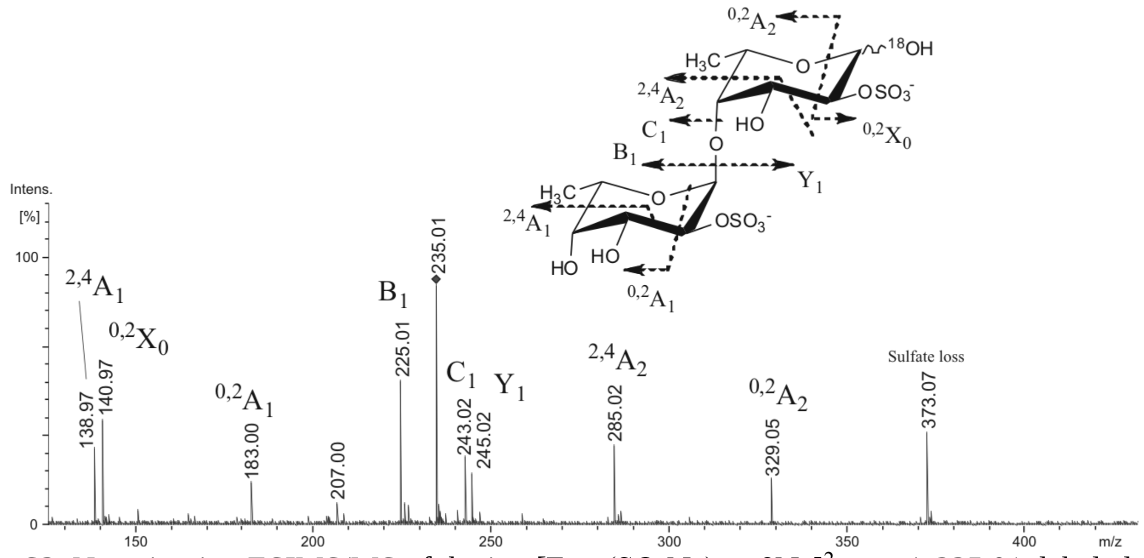

Figure S3. Negative-ion ESIMS/MS of the ion [Fuc₂(SO₃Na)₂ – 2Na]$^{2-}$ at *m/z* 235.01, labeled with ^{18}O

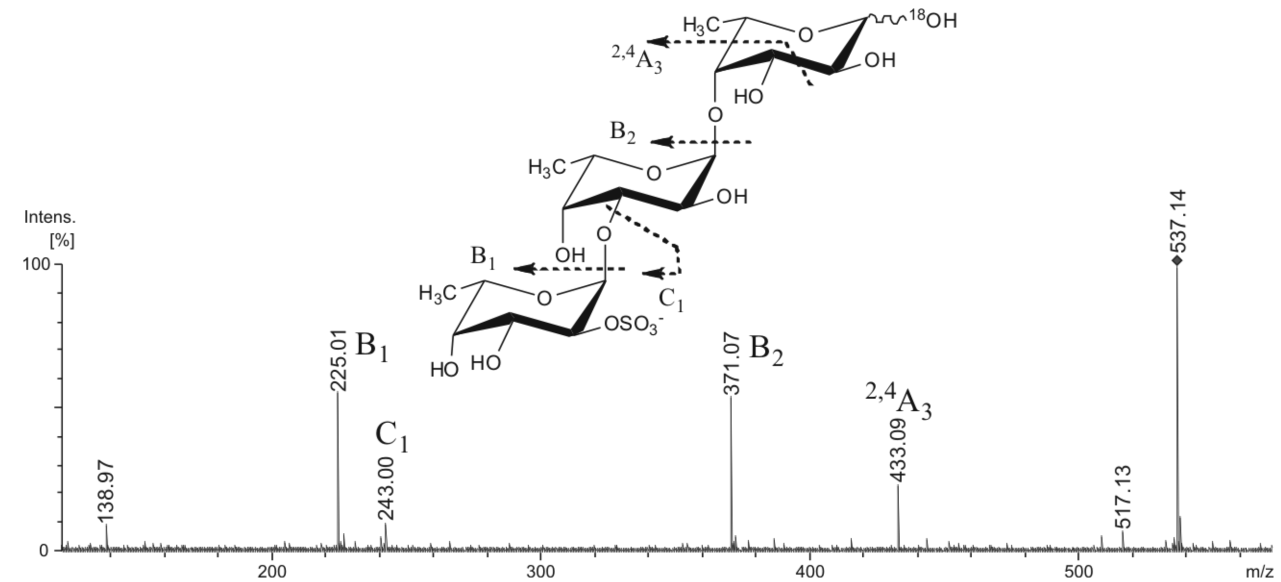

Figure S4. Negative-ion ESIMS/MS of the ion [Fuc_3SO_3Na – Na]$^{–}$ at *m/z* 537.14, labeled with ^{18}O

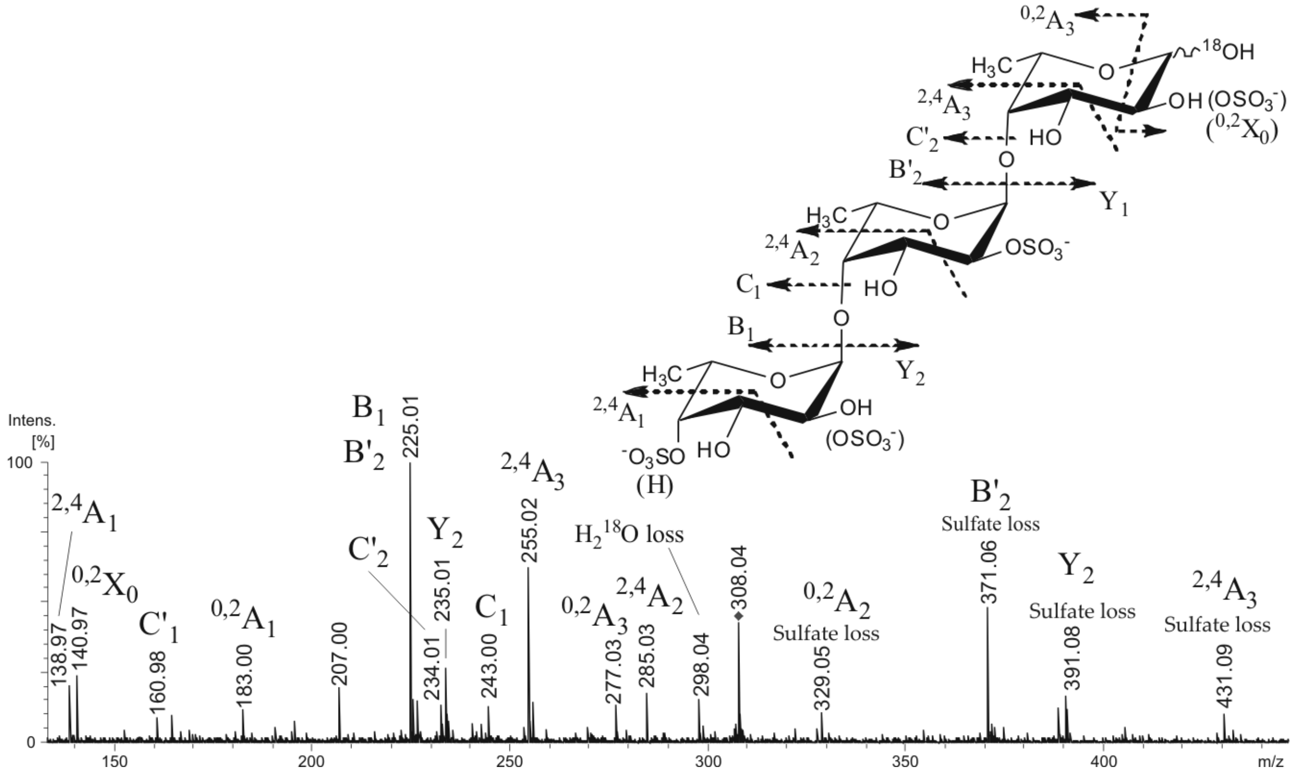

Figure S5. Negative-ion ESIMS/MS of the ion [$Fuc_3(SO_3Na)_2$ – 2Na]$^{2–}$ at *m/z* 308.04, labeled with ^{18}O

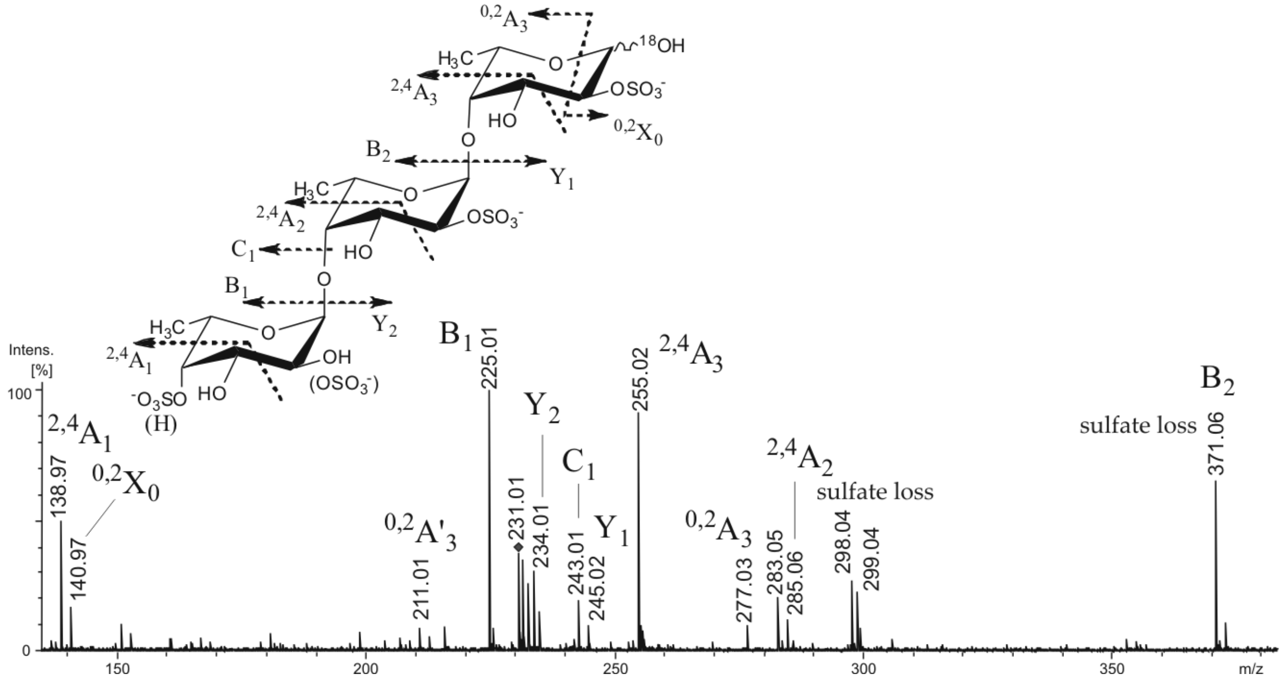

Figure S6. Negative-ion ESIMS/MS of the ion $[Fuc_3(SO_3Na)_3 - 3Na]^{3-}$ at *m/z* 231.68 (with ^{18}O label)

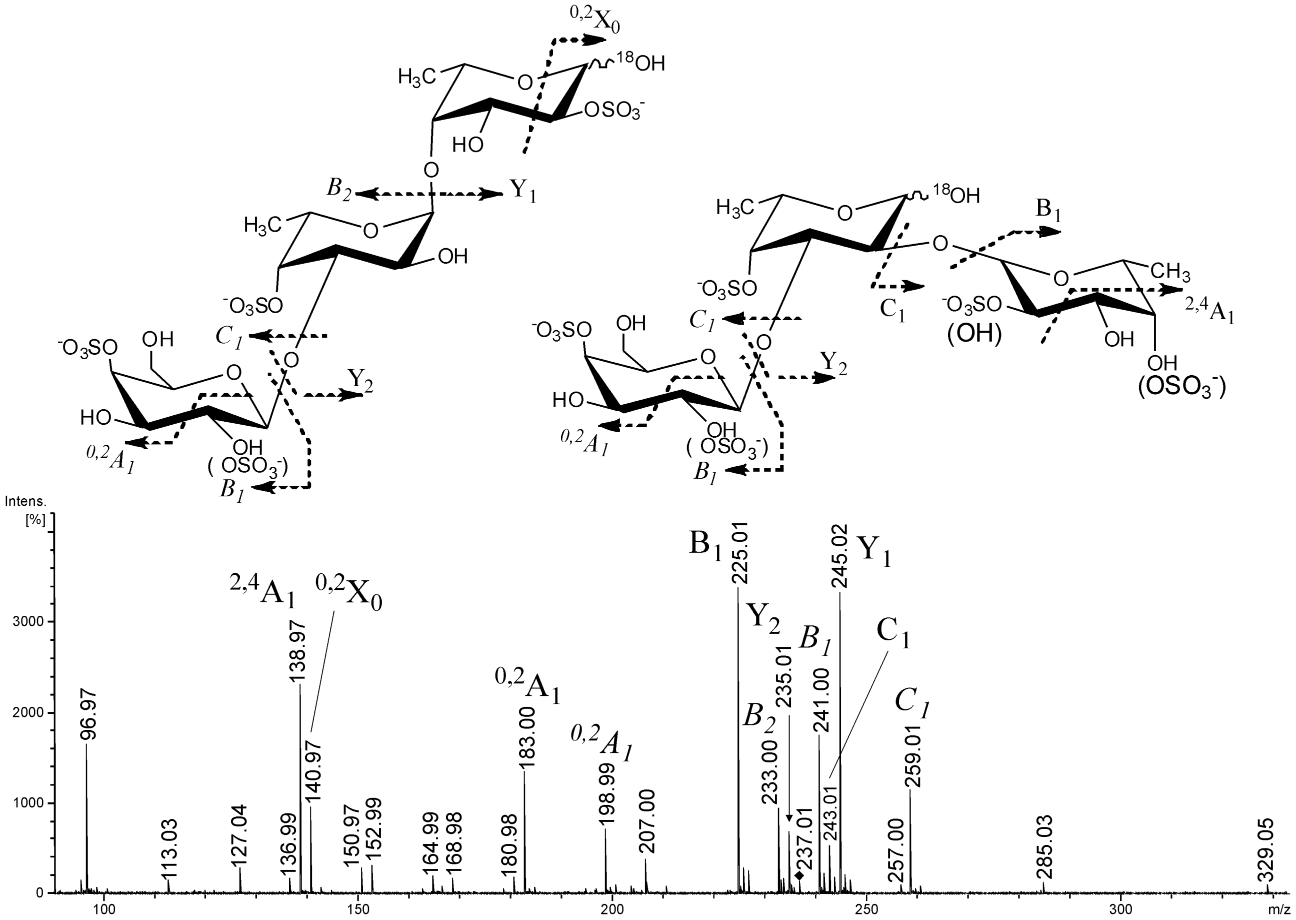

Figure S7. Negative-ion ESIMS/MS of the ion $[Fuc_2Gal(SO_3Na)_3 - 3Na]^{3-}$ at *m/z* 237.01, labeled with ^{18}O

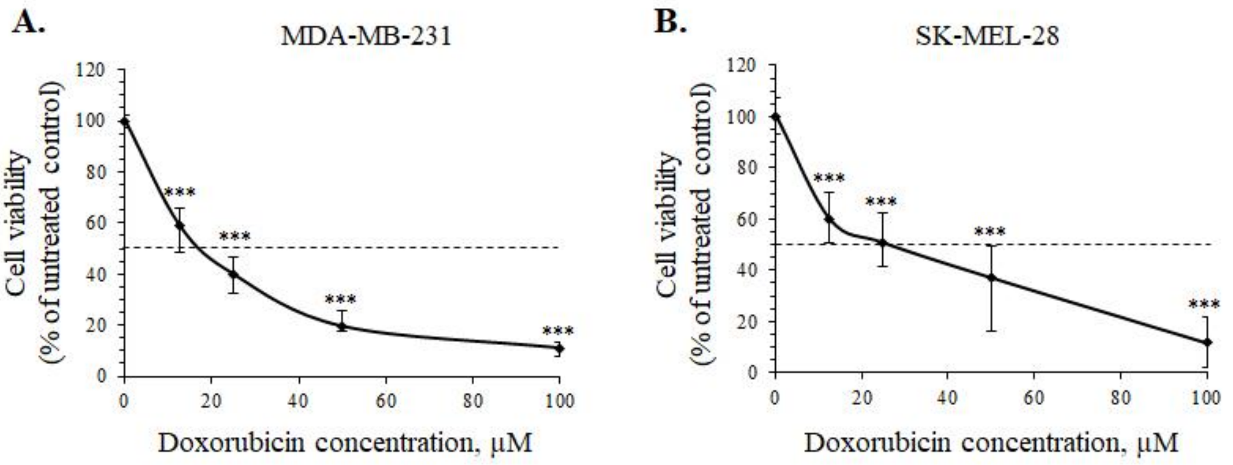

Figure S8. The cytotoxic effects of doxorubicin against breast cancer MDA-MB-231(A) and melanoma SK-MEL-28 (B) cells

MDA-MB-231 and SK-MEL-28 cells were treated by doxorubicin (12.5, 25, 50, 100 μM) and incubated for 24 h. The cell viability was estimated by MTS assay. The data are presented as mean ± SD for triplicate experiments. A one-way ANOVA indicated the statistical significance compared to control (***$p < 0.001$).
